# An Application for Aesthetic Quality Assessment in Photography with Interpretability Features

**DOI:** 10.3390/e23111389

**Published:** 2021-10-23

**Authors:** Fernando Rubio Perona, María Julia Flores Gallego, José Miguel Puerta Callejón

**Affiliations:** 1 Departamento de Sistemas Informáticos, Universidad de Castilla-La Mancha (UCLM), 02071 Albacete, Spain; julia.flores@uclm.es (M.J.F.G.); jose.puerta@uclm.es (J.M.P.C.); 2 Intelligent Systems and Data Mining Research Group, Albacete Research Institute of Informatics (I3A), Universidad de Castilla-La Mancha (UCLM), 02071 Albacete, Spain

**Keywords:** aesthetic, application software, deep learning, fine-tuning, image quality, transfer learning

## Abstract

Automatic aesthetic quality assessment is a computer vision problem in which we quantify the attractiveness or the appealingness of a photograph. This is especially useful in social networks, where the amount of images generated each day requires automation for processing. This work presents **Aesthetic Selector**, an application able to identify images of high aesthetic quality, showing also relevant information about the decisions and providing the use of the most appropriate filters to enhance a given image. We then analyzed the main proposals in the aesthetic quality field, describing their strengths and weaknesses in order to determine the filters to be included in the application **Aesthetic Selector**. This proposed application was tested, giving good results, in three different scenarios: image selection, image finding, and filter selection. Besides, we carried out a study of distinct visualization tools to better understand the models’ behavior. These techniques also allow detecting which areas are more relevant within the images when models perform classification. The application also includes this interpretability module. **Aesthetic Selector** is an innovative and original program, because in the field of aesthetic quality in photography, there are no applications that identify high-quality images and also because it offers the capability of showing information about which parts of the image have affected this decision.

## 1. Introduction

One of the most important and active fields in the scientific community is computer vision due to its large number of applications in different domains. In recent years, deep neural networks [1] have enabled solutions to computer vision problems that, until recently, seemed unapproachable. This scenario has caused more complex tasks to emerge such as the automatic assessment of aesthetic quality in photography. However, from a practical point of view, few solutions have been presented that make use of the research developed on aesthetic quality, which motivated this work.

The concept of aesthetic quality in photography refers to those image properties that make pictures attractive or pleasant for most people. These properties include applied filters, the harmony of colors, etc. All in all, determining aesthetic quality is one of the most complex problems within the field of computer vision due to the subjectivity of the task, since the opinion of two individuals about the quality of a single image can be completely different. Even among expert photographers, there may be distinct judgments. Notice that we should not confuse this aesthetic concept with the quality of an image in terms of resolution.

Owing to the large number of images that are continuously generated, aesthetic quality assessment has attracted increasing interest in the scientific community. In the case of social networks, such as Instagram, Google Photos, or Flickr, there are more than 600 million daily active users who upload pictures. Therefore, if those platforms are able to sort or filter the images according to their aesthetic quality, it could be extremely helpful and profitable. The automation of this task results in some remarkable applications such as organizing image albums based on their quality, especially useful for websites such as Flickr (https://www.flickr.com/, accessed on 19 October 2021) or Unsplash (https://unsplash.com/, accessed on 19 October 2021). However, it can also be used for filter recommendations or even for online assessments in a camera, to improve the quality of the photographs we take. Even advertising can benefit from aesthetic quality applications by means of the creation of more attractive images for commercials and marketing.

For these reasons, in this work, an app called **Aesthetic Selector** is developed, which is able to evaluate the aesthetic quality of photographs. The application is divided into two tools, a filter, which selects the best images in an input folder, and an explainer, which identifies the regions of the photograph that have more influence on classification. The filter tool was combined with other applications to obtain different results demonstrating great success.

In order to carry out the development of this application, different challenges were tackled. The first one was how to determine a suitable image quality benchmark: the subjectivity of the aesthetic quality assessment requires us to take into account individual ratings, which represent multiple reviews about how good/bad a photograph is; this information must be summarized, synthesized, and compressed in order to create interpretable values of the image quality. These ratings are normally limited to a range where the maximum and minimum values represent an absolute belief about how high or low the quality of the picture is, respectively, whereas the intermediate values represent uncertainty and doubts about that image attractiveness.

The most common approach to synthesize that users’ rating information consists of obtaining an automatic classifier able to determine if the image is good or bad. This procedure transforms the information into a binary classification, where the images are labeled as “snapshot” (bad quality) or “professional shot” (good quality). In addition, some classification techniques not only report the label, but also a degree of belonging to each class. For instance, an image could be classified with a 59% degree of belonging to “professional shot” (and 41% to “snapshot”), but another one could be classified with a 90% degree of belonging to “professional shot” (and 10% to “snapshots”); both will be labeled as good ones but, there will be a big difference between them. These degrees try to be close to represent real personal opinions.

In this article, deep learning is used to solve the aesthetic quality assessment task. More specifically, this work is focused on transfer learning [2], which is based on using pretrained deep neural networks from other datasets where more information is available. The most widely used deep neural network structures for computer vision are AlexNet [3], VGG [4], Inception [5], ResNet [6], and MobileNet [7], and all of them have arisen from different research works on the ImageNet task [8]. Then, the main objective of using transfer learning is to take advantage of all the knowledge generated by the learned networks from the ImageNet task, which are capable of identifying 1000 different image concepts.

Deep learning models are considered as a black box, as it is not possible to explain their internal behavior. However, in recent literature, many articles and research works have proposed distinct tools able to visualize this internal functioning. These tools attempt to show further information when these models generate a particular prediction. One of the most important challenges of this work is the use of these novel techniques in the problem of aesthetic quality assessment and its integration in the application we present.

Before an in-depth description of our application **Aesthetic Selector**, an analysis of the most relevant aesthetic quality assessment models, together with visualizations tools for deep neural networks, is carried out. Having said that, the paper is organized as follows. The state-of-the-art literature is given in Section 2. Next, in Section 3, our proposals based on transfer learning are presented. Section 4 presents an overview of the interpretability tools we considered. Section 5 describes our desktop application and its applicability. Next, Section 6 provides a general discussion of the results and the solution used in the app. Finally, this paper is concluded in Section 7, summarizing the main contributions and future research lines of research.

## 2. Related Work

When dealing with aesthetic quality assessment in images, the end user expects to see information in the form of labels such as “snapshot” or “professional shot”. Furthermore, the user may want to see a rating such as, for example, Amazon stars, where zero stars represent a very bad image and five stars indicates that the photo is wonderful; or even more specific values such as a scale from one to one-hundred. However, the aesthetic assessment is a highly subjective task, so the information that end users expect is not directly within our grasp. Usually, the image feedback is a set of ratings representing the annotators’ perception of the aesthetic quality, i.e., their belief that an image is good or appealing. In fact, images have multiple values—one from each annotator—forming, thus, a distribution.

**Figure 1:**
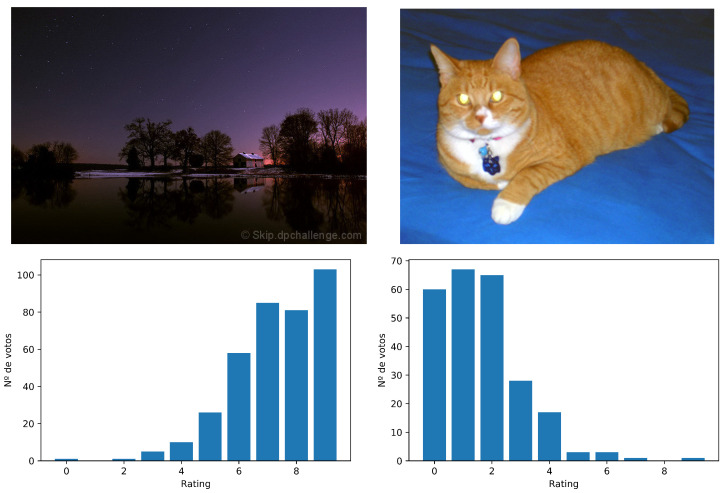
Example of one professional image (**above**, **left**) and one of poor quality (**above**, **right**). In the lower part, the distribution of the votes for each image is shown.

Information on aesthetic quality is often found on professional photography sites such as DPChallenge or Photo.net, where different photographic challenges are proposed based on a theme and users upload and evaluate different photographs. However, these pages only offer information about the number of votes, not the user that rates the image. Figure 1 shows two images of DPChallenge with the different ratings received in the range [1–10]. Each vote in the high values represents a user’s belief that the photo has a high quality, while a vote in the lower values represents a belief that the image is of poor quality.

**Figure 2:**
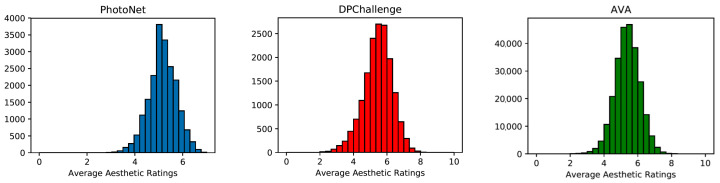
Distributions of the average scores received through three well-known datasets.

Diverse databases have been generated from these sources for the problem of aesthetic quality. Table 1 shows the three most broadly used datasets among them. The number of images they contain is also indicated, as well as the information of the ranges in which the images have been evaluated. In Figure 2, we see the vote distribution of the images of each of the databases, where in all cases, we see a clear normal distribution with the average close to five.

In [9], the PhotoNet and DPChallenge datasets were mentioned as a subset of images obtained from their respective web pages.

The dataset AVA [10] was created by extracting a much larger set of DPChallenge. Each image has a mean of 200 votes from different users. In addition, each image is labeled with the type of challenge in which it was uploaded, the photographic style (if any), and certain labels on objects that appear in the image. In Table 2, there is a summary of the number of images by their mean and the number of votes.

The first attempts to solve this problem were based on transforming the ratings assigned to an image into certain statistics that allow us to convert the problem into a binary supervised classification one. For example, we can use metrics such as the mean or the median to separate the images into “snapshots” or “professional shots”. Then, a threshold is set at the midpoint of the ratings range, that is if the votes go on a scale of one to ten, the cut point is set to five. Finally, the average is obtained for each image $\mu_{i}$ from their votes and compared to the threshold. In Figure 1, in the case on the left, we have μ=8.31 and on the right μ=2.62. When having a range of ten possible values, the threshold is placed at five, so the image on the left would be classified as “professional shot” and the one on the right as “snapshot”.

Both [11,12] proposed to solve the problem with low-level handmade features to try to identify more complex properties of the photographs and to be able to separate both categories. However, these proposals were soon overtaken by general feature extraction techniques, such as the descriptor GIST [13] or scale-invariant feature transform (SIFT) [14,15,16].

The arrival of AVA also proved that the proposed solutions were only useful in the environment for which they were trained, and they failed in a realistic environment. In [10], it was also mentioned that many works utilized a parameter δ to remove those images close to five. This was done not only in the training set, but also in the test set, which makes the problem easier, but further from reality. For example, in [17], we find a comparison between hand-crafted features within the AVA problem domain, where the proposed features outperform the general image descriptors. Nevertheless, this research work only employed 20% of the whole AVA dataset, which simplifies the original task. For this reason, we used as a basis those works in aesthetic quality assessment focused on resolving AVA with the full set of images, because such proposed solutions improved the results in all the tasks involving aesthetic quality.

The AVA dataset not only outlined a more complex problem, but it also facilitated the use of deep learning models. In [18], one first deep learning model with seven layers obtained 3% better accuracy than the results in AVA’s paper. Besides, they were able to create distinct enhancements to reach 6% improvement.

However, more recent research works such as [19] showed the possibility to obtain more reliable results than those presented up to that moment, by performing the process of *fine-tuning* in the last layer of the AlexNet and VGG models, which obtained better results than AVA’s paper, offering an improvement of 10%.

More recent works have proposed alternatives to binary classification, as it is feasible to use all the information of the votes from the datasets and not only the mean of them. Such is the case of NIMA [20], which tries to directly predict the distribution of votes through *fine-tuning*. This work proposed an output layer with as many nodes as different ratings can be assigned to the image, 10 in the case of AVA. Each node represents the distribution of votes in the image, and their sum is one, allowing using the *softmax* layer as the activation function. Finally, NIMA uses the Earth Mover’s Distance (EMD) [21], which accounts for the cumulative probability distribution to calculate the loss function.

### Interpretability in Aesthetic Quality

Interpretability in machine learning models is one of the most important topics currently. We could say that a particular classification is interpretable if we (humans) can comprehend how certain decisions or predictions have been made. When applying this concept to computer vision with (deep) neural networks, class activations maps (CAMs) [22] are the most widely used tools. A class activation map for a particular category indicates the discriminative image regions used by the CNN to identify that category. The class activation map is a weighted linear sum of the presence of visual patterns at different spatial locations. By upsampling the map to the size of the input image, the most relevant regions with respect to the particular category within the image are detected.

Later, more sophisticated methods were designed. Gradient-weighted class activation mapping (Grad-CAM) [23] uses the gradients of any target label, flowing into the final convolutional layer to produce a coarse localization map highlighting the important regions in the image for predicting the concept. Grad-CAM is applicable to a wide variety of CNN family models without architectural changes or retraining. Grad-CAM++ [24] improves by providing better visual explanations of CNN model predictions. This improvement is in terms of better object localization, as well as explaining the occurrences of multiple object instances in a single image. They used a weighted combination of the positive partial derivatives of the last convolutional layer feature maps with respect to a specific class score. Furthermore, there exist CAM approaches that are gradient independent. For instance, Score-CAM [25] obtains the weight of each activation map through its forward passing score on the target class, the final result being obtained by a linear combination of weights and activation maps.

There exist few works where interpretability was applied to aesthetic quality in image. For example, Reference [26] showed the training of a neural network where some global average pooling (GAP) elements were added to extract CAMs. These maps are used to obtain aesthetic sections over the images. In summary, they proposed an automatic technique for image cropping, which is based on the aesthetic map and gradient energy map. In [27], they extracted the class activation maps from the ImageNet models in the original paper, using those labels as the target. Then, a new model was created, which combined two inputs: the original image and the activation map. This allows the model to focus on the most relevant object within the image. Another strategy was developed in [28], where first, clustering is applied for identification of image features and, then, distinct quality-based classifiers are obtained given the label. The work described in [29] is closer to our approach. They obtained two CAM-based models, which also used *fine-tuning* on the AVA dataset to later apply them in a cropping application.

## 3. Materials and Methods

Firstly, we performed a study of the distinct transfer-learning-based alternatives. As we saw in Section 2, the best proposals currently are based on applying this technique on pretrained networks. In this work, different solutions based on two main transfer learning techniques are presented and analyzed.

### 3.1. ConvNet Features

**Figure 3:**
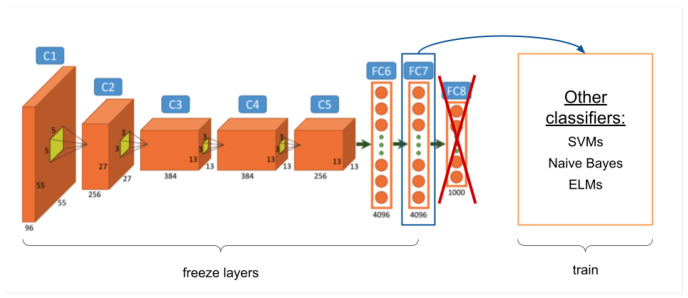
*ConvNet features* extracted from the last hidden layer are used to learn a new model.

The first of the transfer learning techniques can be seen in Figure 3 and consists of the extraction of the characteristics from a neural network, which are known as ***ConvNet features*** or ***DeCAF*** [30] (hereafter *ConvNet features*). This technique removes the last layer(s) of a pretrained network to obtain the activation of the desired layer as the output. In this case, the network is not retrained, since its weights are frozen. The activations in the previous layers are obtained to be used as *input* values in other models such as Bayesian models [31] or support vector machines (SVMs) [32]. Therefore, this first proposal studies the performance of the different layer activations, applying them to the aesthetic quality assessment as a binary classification.

When an image in a neural network (*forward* process) is evaluated, not only the output values are obtained in each layer of the network, but a series of activations is also generated. This information can be used as a descriptor of the image, since they have great descriptive capacity and are generally used in problems in which there are not enough data to train a neural network. These characteristics are extracted from pretrained networks designed for a specific task, and then, they are used to train models to solve similar challenges. In this work, and in most image problems, the pretrained networks were obtained from ImageNet. The last layers of these neural networks are close to describing 1000 different concepts, and this information can be used as a very complex set of image characteristics.

*ConvNet features* are a popular technique in computer vision. However, in the particular task of aesthetic quality, they have been quite superficial. In [33], the FC7 layer from AlexNet in an SVM model was used as the image descriptor, which provided the best results compared to any other image descriptor. Features extracted from distinct networks were also used in [34,35,36], but these studies only used a reduced portion of AVA. In [37], a study of the performance of *ConvNet features* extracted from two neural networks (AlexNet and ResNet) in the evaluation of aesthetic quality was carried out.

This work showed that the use of these features outperforms other standard descriptors. In order to complete this study, the most popular networks in computer vision were included: VGG16, Inception, MobileNet, and ResNet. From them, a set of characteristics were obtained, and these extracted features served as the input for a set of representative classifiers.

Therefore, in all the experiments of this section, the next-to-last layer weights of the network were extracted, as can be seen in Table 3. This table shows the number of characteristics obtained in each case. Models learned from these characteristics are binary classifiers, such as: the SVMs, widely used in image processing; naive Bayes, with continuous variables modeled as Gaussian conditional distributions; and extreme learning machines (ELMs) [38], algorithms based on neural networks that have proven their effectiveness in many problems.

#### Experimentation and Results

Different experiments were carried out using the **full set** of images from the AVA database partitioned into training and testing following previous works, the latter being about 20k images and the remaining 230k for training. The mean was obtained from the votes for each image *i*, and it was labeled as “snapshot” if $\mu_{i}<5$ or “professional shot” if $\mu_{i}\geq 5$. From the AVA images, 5 sets of characteristics were obtained from 4 pretrained networks, as is shown in Table 3. Continuous naive Bayes, SVMs, and ELMs were trained with each set, and their results are reported in terms of accuracy, balanced accuracy, and the AUC.

For the implementation, Keras [39] with the TensorFlow [40] backend was used to carry out the process of *ConvNet features* extraction. The pretrained weights were the Keras defaults. The features extracted were used in different classifiers from SciKit Learn [41].

All the obtained results are shown in Table 4. Naive Bayes is a particular case, where it showed the best results with MobileNet characteristics and the worst ones with VGG16. This was due to the complexity of the descriptor, MobileNet having 1024 features and VGG16 4096. As the MobileNet characteristics beat those results obtained with general descriptors and hand-crafted features, it is also true that their performance is not as good as *fine-tuning* processes. Anyway, in those cases where a neural network cannot be trained, the use of these descriptors should be considered for the problem of assessing aesthetic quality. The best combination is naive Bayes together with MobileNet, which provides a high AUC value. This is quite convenient as MobileNet is one of the simplest and fastest models in deep learning, while naive Bayes also presents two advantages: simplicity and great speed in classification time.

### 3.2. Fine-Tuning

This experiment replicated some of the most relevant models and also offered results with several configurations, different from those presented in the original papers. This is helpful in order to provide the best solutions as the core for **Aesthetic Selector**. This work mainly focused on the *fine-tuning* techniques, since in recent years, they have provided the best results in the aesthetic quality assessment problem.

**Figure 4:**
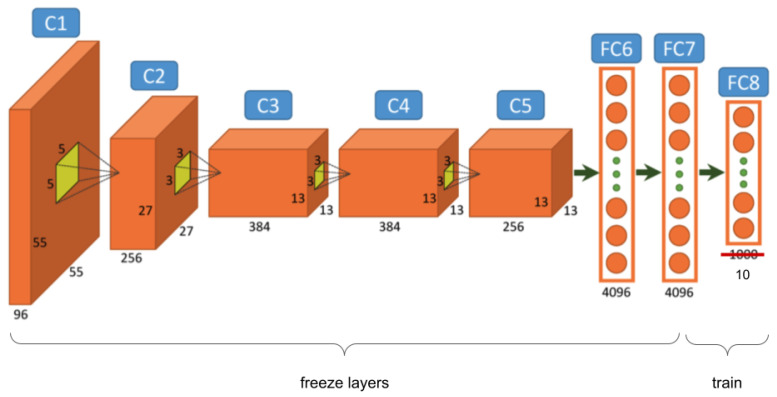
*Fine-tuning* process, where the last layer of a pretrained network was modified and whose weights would be learned. The rest of the layers do not suffer modifications.

*Fine-tuning* is a technique of transfer learning, which consists of adapting the structure to the task and retraining the changed connections of the network with the available data. It is possible to fine-tune the rest of the layers of the ConvNet or to keep some of the first ones frozen. In Figure 4, we can see an example of *fine-tuning*, where all the layers are frozen except FC8, which was modified for a 10-class problem, and their weights were reset. Only this last layer was trained with *fine-tuning*.

From all the state-of-the-art proposals, 4 models where selected:

- DAN1 [19] is a binary classification model obtained from the fine-tuning process. In this case, the structures of all layers of the model, except the *output*, are the same. However, we believe that this technique can be used more effectively by applying modifications to the previous layers, as long as the model allows it. Fully connected layers from AlexNet and VGG16 were designed for a 1000 output problem, and the complexity of these layers can be reduced to a binary output problem. These layers contain most of the parameters, and our proposal reduces significantly the size of the network without performance loss;
- A-Lamp [42] is a regression model with a very complex structure based on select patches from the image. A re-implementation of the model was developed, but the complexity of the structure and the lack of information of different hyperparameters makes this re-implementation’s results worse than those presented in the paper;
- NIMA [20] is a fine-tuning model that predicts the votes’ distribution using EMD as the loss function. In this work, we explored different network architectures apart from that presented in the original paper;
- The Bernoulli distribution network (BDN) is proposed based on [43]. Instead of having for each image the labels “professional shot” or “snapshot”, pictures are represented by a probability distribution [θ;1−θ]. In this way, the probability of being a “professional shot” is θ and the probability of being a “snapshot” is 1−θ. θ is estimated as the average of the normalized votes, so it is a probabilistic label instead of a categorical label. In this case, there is a unit softmax output in the networks, representing the probability of being a bad shot or a good shot. The only change in the training phase is the loss function used, where the actual probability is compared with the true probability of the image. We opted to use the mean squared error (MSE). (Similar results were obtained with the entropy loss. This is because both errors use the differences between parameters when the gradient is evaluated).

It should be noted that most of the initial research works suffered from a problem with the evaluation, as indicated in [37], since the success rate or *accuracy* was the only metric used in many proposals when validating their models. Note, for example, that in the AVA dataset, this metric is not very informative. If we binarize the class from the votes, taking 5 as the threshold (since the range goes from 1 to 10), we observe an imbalance of the class, where 70% of cases are “professional shots” and 30% “snapshots”. In this case, reporting a hit rate of 70% has no added value, since all the images can be classified as good, and we could easily obtain the same performance rate.

Different metrics have been proposed to overcome those problems and avoid inaccuracies and the unnecessary loss of data, as in the research works [19,20,37]. Some of the classification metrics found are *balanced accuracy*, which reports the success rate for each of the classes, and the area under the curve value (AUC), which also relates the positive true rate to the positive false rate. Most of these models also return a score of the prediction. From these values, the MSE, the Spearman’s rank correlation coefficient (SRCC), and Pearson correlation coefficient (PCC) are obtained. The last two measure the relationship between two datasets; in this case, the ground truth and the predicted distributions. Finally, in those models that return the distribution, the EMD is also reported.

#### Experimentation and Results

Experiments were carried out for five deep neural network structures: AlexNet, VGG16, ResNet, Inception, and MobileNet. All pretrained models were from the Keras default configuration, except AlexNet, where its weights were taken from caffe model zoo (http://caffe.berkeleyvision.org/model_zoo.html, accessed on 19 October 2021). In all cases we implemented a *fine-tuning* process modifying the output layer and retraining the weights of all layers with an Adam optimization function, a learning rate of 3 × 10^−6^, and 20 epochs. These structures were retrained with the full set of images from the AVA database partitioned into training/testing (230k/20k images), following the most recent works. For this work, we used Keras with the TensorFlow backend to carry out the process of *fine-tuning*. All this was run on a Tesla K40c GPU.

In the DAN1 cases, the network structures selected were AlexNet and VGG16, since they are the same as the original paper and they allow modifying the FC6 and FC7 layers, besides the “output”. Four different size values were tested: 4096 (default), 1000, 500, 250. In Table 5, we can see the number of parameters of each network and the memory they require. In the other three proposals, the pretrained networks for fine-tuning were ResNet, Inception, and MobileNet.

Table 6 shows all the networks, where most of them were obtained from the fine-tuning process (except Murray and Rapid).

In terms of classification and due to the imbalance problem, DAN1(VGG16) seems to be the best model if we look at balanced accuracy. However, this model has a considerable size, its use not being possible in limited devices such as smartphones, digital cameras, or IoT equipment. In fact, the model size affects the development of a cloud solution or a desktop app. For this reason, the reduction of the layers proposed is very important. The values in Table 6 demonstrate that the reduction did not affect the results, with a significant memory savings. In DAN1 (VGG16), the results were almost identical in all networks, regardless of the size of the dense layers. It should be noted that only the *accuracy* was affected, and the network with dense layers of size 500 worked better than the original with 4096 layers.

With respect to the other three proposals, there was hardly any difference between the results, perhaps the A-Lamp approach being the worst one, mainly due to the lack of hyperparameters in the original paper, as we mentioned before. Regarding the structures, our NIMA and the original one worked better with Inception, as well as our Bernoulli implementation. All in all, the results for Bernoulli and NIMA seemed to be the best in general terms. Our implementations were close to this results, and in some of the metrics, they even outperformed the original ones.

In addition to the implemented models, we carried out a series of experiments where we performed the same *fine-tuning*, but reducing the number of training images with two models, our Bernoulli and our NIMA (both with the Inception architecture). As *fine-tuning* is a retraining process, we wanted to check if it is possible to perform this process with a smaller number of images. The results are shown in Table 7, and the conclusions are clear: currently, *fine-tuning* obtained the best results when the entire AVA database was used. This led us to think that for the *fine-tuning* to be successful on this aesthetic quality problem, it is necessary to have information from as many photographs as possible. It should be noted that although the output of this problem does not have as many nodes as the ImageNet task, the input is much more varied, since the images are very different from each other, so the more input images, the better this *fine-tuning* will be. This also indicates that it is possible to further improve this process if more images were available for training. This would also explain why the models from scratch do not perform as well, since if it is still possible to improve the fine-tuning process by increasing the number of images, it is obviously necessary to have more images to increase the performance of the networks learned from scratch.

Finally, between our NIMA and our Bernoulli, the second one was selected since Bernoulli’s output is composed of two classes representing “snapshots” and “professional shots”, which makes this model perfect to analyze with interpretability tools.

## 4. Interpretability

When interpretability is addressed in the deep learning paradigm, it mainly refers to the comprehension of the behavior provided by the deep neural networks when processing an instance, but also to the comprehension of the role of the intermediate layers within the network.

A great variety of tools can be found in this area when using ConvNets. These tools attempt to understand the behavior of both the convolutional layers and the fully connected ones. As described earlier, in the particular aesthetics case, some of these techniques have also been applied, mainly CAM, but typically they are focused on just making (automatic) crop operations with the most relevant information.

The current paper aimed at studying further how the implemented models behave when using two distinct interpretability methods: smooth grad saliency and GradCAM++. When developing this work, CAM, ScoreCAM, and vanilla saliency (also known as the saliency map or sensitivity map) were also included in the tests. Finally, only those techniques that seemed to contribute more clearly were selected.

A saliency map represents the pixel influence within an image when performing classification. This influence or strength is computed by using the difference provoked in the class when that pixel value is varied, even with small variations. With this technique, those regions closer to the pixels significant for the class become also significant for proximity reasons. This is why the resulting plots could be perceived as noisy. This motivated smooth grad saliency, where several neighbors of the input are generated by Gaussian-based perturbations, and the resulting saliency map is, in fact, the average of the saliency maps from all the neighbors.

On the other hand, CAMs have shown the limitation of their necessity from global average pooling (GAP) layers to provide good results. However, it is complex to use CAMs in the aesthetics field given the great variety of models and structures this problem poses. For this reason, GradCAM, a CAM variation designed to avoid those GAP layers, is preferred. On top of it, we included an improvement called GradCAM++, whose results are reported. GradCAM++ basically compares the positive partial derivatives of the last convolutional layer feature map with a specific class score, such as weights, to generate a visual explanation for the corresponding class label.

From all the implementations shown in Section 3.1, the best results were obtained with Bernoulli and NIMA, both of them using Inception as the basis. From these two models, we determined that Bernoulli is more interested as it has a binary class, whose values can be considered as good/bad quality. The behavior of both smooth grad saliency and GradCAM++ within this model was analyzed, trying to catch the first impression of how they relate to the images. The results obtained in this respect were directly taken from the application **Aesthetic Selector**.

### 4.1. Smooth Grad Saliency

**Figure 5:**
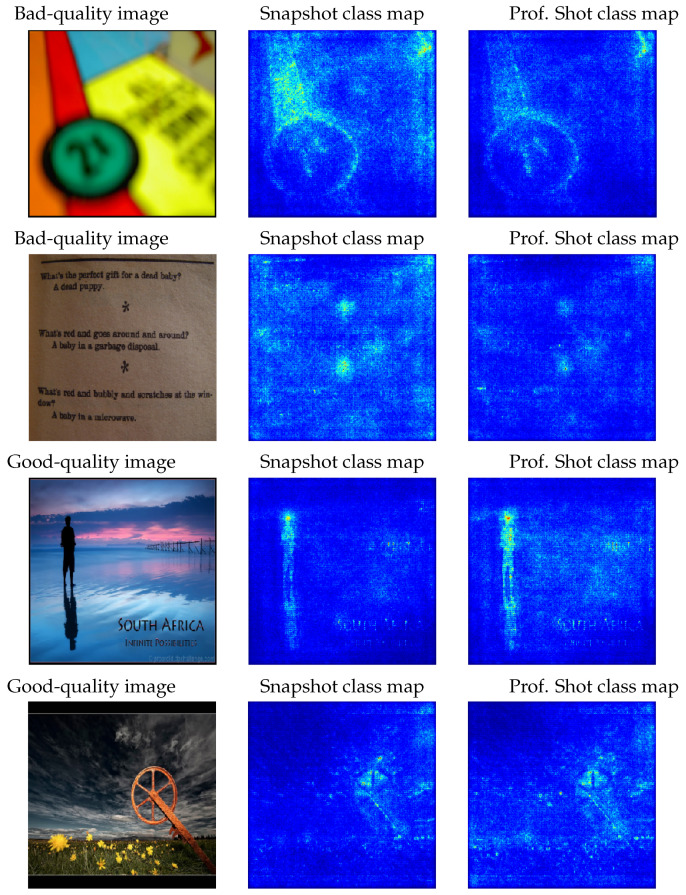
Example of two poor-quality (top first rows) and two professional images (bottom two). Next to them, we see the application of smooth grad saliency for the bad and good classes.

Figure 5 presents some examples from the AVA dataset together with the smooth grad saliency maps obtained. From them, we can observe which elements are the most influential when classifying. There will be more or less active pixels depending on the image quality and the associated value of the class. However, it can easily be seen that the main elements are shared in both class labels. This is due to the fact that Bernoulli maintains a strong relation between both classes. If one class receives a positive activation, it also affects, in a reverse way, the other class. It would be really interesting to study those pixels that uniquely affect one class or the other, but also including the type of such influence, that is if it is negative or positive with respect to the class.

**Figure 6:**
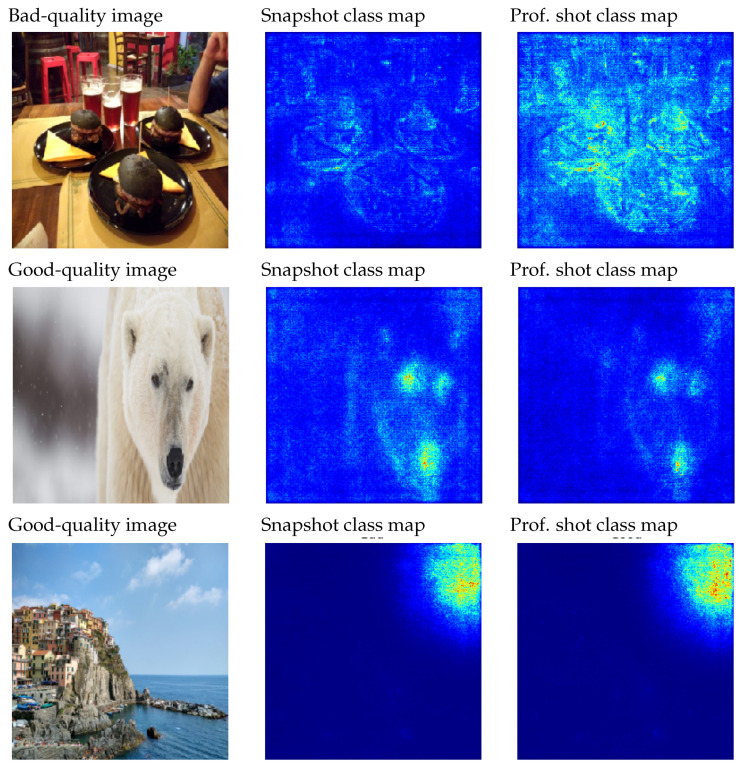
Three selected images: one of poor quality (top) and two of professional quality, shown in rows. Next to each other, we show the smooth grad saliency for the bad and good class.

Identification of the most significant areas in the image when performing classification is of particular importance, especially because the goal is to improve the aesthetic quality. Figure 6 contains some examples where the saliency map did not work as would have been expected. On the top there is an image classified as bad quality, whose saliency maps contain the majority of dots in the category “professional shot”. The opposite case can be found in the middle image. In the case where the images show a portion of sky (example at the bottom), it seems that the model is able to decide by simply considering those border regions.

### 4.2. GradCAM++

**Figure 7:**
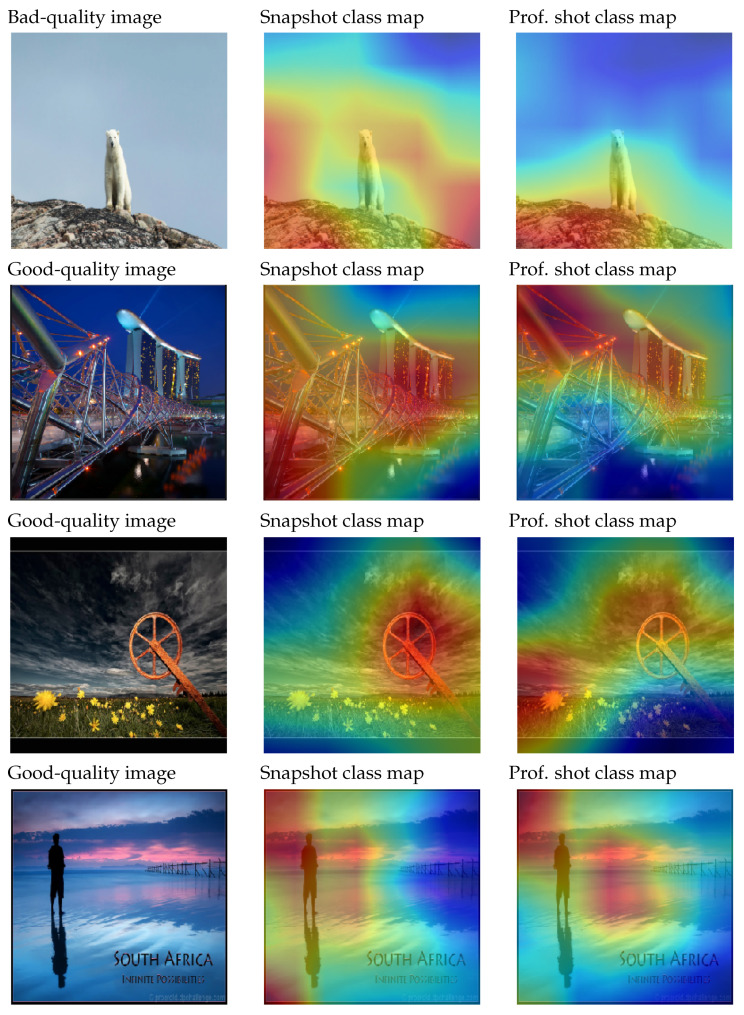
Example of a poor-quality image (top image, bear) and three professional images (the rest). Next to them, we see the application of GradCAM++ for the bad and good classes.

Figure 7 shows some images with a distinct quality level. Those heat maps with more intensity correspond to good quality. Furthermore, it is interesting to analyze those areas that present various intensity levels. This indicates that certain areas within the image present simultaneously features of good and bad quality. For instance, it could happen that the main element of the image is correctly focused and the color composition is correct, but the element is not satisfactorily located in the image. However, the different sources (reasons) for detecting the good/bad areas cannot be perceived just with the given information. We need to remark that only the areas that contribute positively are shown with this technique. This implies that those elements that contribute only negatively to a class and do not contribute positively to the other one will not be shown.

## 5. Aesthetic Selector App

The models learned in this work, as well as the visualization tools analyzed aimed at the development of an application, **Aesthetic Selector**. As shown in Section 3.2, the models that offered the best results were those of *fine-tuning*, specifically NIMA and Bernoulli. Both offer good results, but as previously done, we chose the Bernoulli model as the core of the application. This decision was based on its capability to provide a direct output, and, since the interpretability tools analyzed were based on a binary classification the application should use the same model with which image predictions are made. This section presents the application resulting from the current work and some examples of use.

**Aesthetic Selector** is a desktop app that evaluates different images, giving them an aesthetic quality score. Then, the app obtains a rank based on the aesthetic score, where the best-rated images are at the top and the worst at the bottom. Finally, the application returns a subset of the best images, each one labeled with the score obtained by the app. The size of this subset is based on a percentage the user can previously select.

This way, the user has to select the images’ source folder, a percentage of images to keep, and finally, the destination directory for the subset of images filtered by the application. The percentage of images is applied to the number of images detected in the source folder, e.g., if in the source folder, the user has 1000 images and he/she select 10%, the app will return the 100 best images, in terms of aesthetic quality, and will store them in the destination directory.

This application was designed to identify the best images in terms of aesthetic quality at both the professional and user level. It has been used by photographers as a previous filter, going from having to evaluate thousands of images to only hundreds and, consequently, saving a considerable amount of time.

**Figure 8:**
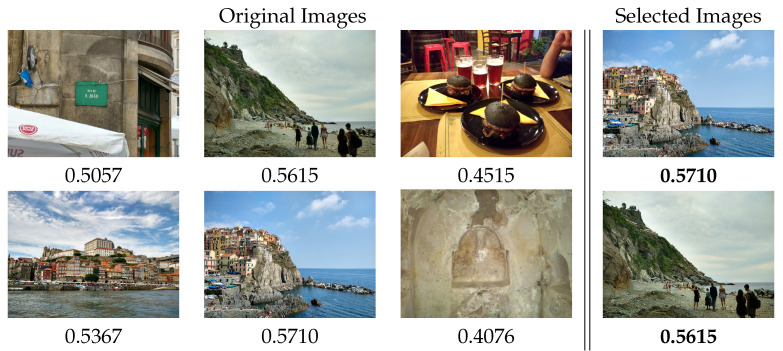
Subset of images from the main author library and the images selected with the application.

At the user level, the application selects those images with the greatest aesthetic quality score, which allows the user to obtain an album of the best photos. In Figure 8, a subset of images can be seen, and although all of them are professional, the application is able to identify those with better quality. Some of these images were finally uploaded to social networks such as Flickr and Unsplash.

It is true that, in both cases, the final decision belongs to the user, since the choice of the most attractive photos, especially in the case of social networks, is very personal and highly subjective. However, the previous filtering offered by the application makes this selection task much more bearable and effective, as we will be able to focus on the most attractive photos, especially in the case of photographers, because the selection process, when the number of images is high, can be affected by the fatigue of reviewing the images one by one.

**Figure 9:**
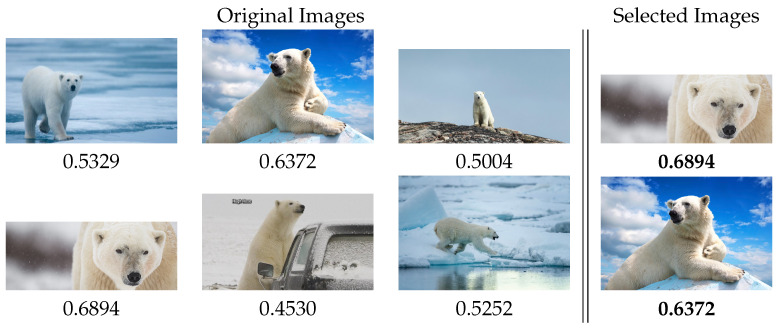
Subset of images from the Google “Polar bear” image search and the images selected with the application.

**Aesthetic Selector** can be combined with different tools to create a more complex, useful, and versatile application. First, it was used in conjunction with Google image download tools such as the google-images-downloader (https://github.com/hardikvasa/google-images-download, accessed on 19 October 2021) library. The goal was to obtain a tool capable of searching the most attractive images based on keywords. For example, the words “Polar bear” were used to download the images related to that term, obtaining more than 300 images. Then, the application selected 20 images related to “Polar bear” with good aesthetic quality. An example of this search can be seen in Figure 9.

The possibilities of combining an image search engine together with **Aesthetic Selector** are innumerable. Anyone who requires high-quality images to, for example, make a business website, create a product catalog, or make any advertising banner can benefit from this tool.

**Figure 10:**
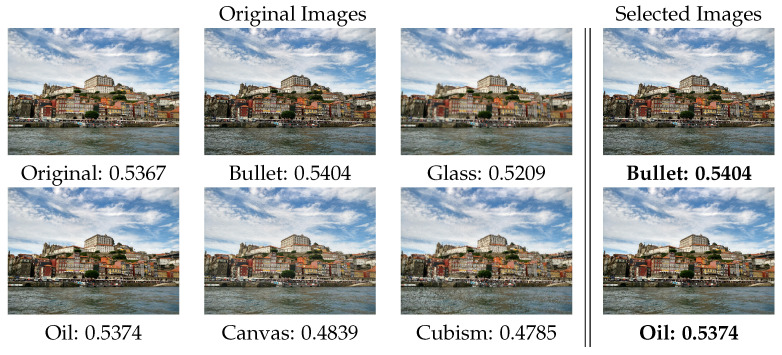
Subset of images from applying GIMP’s filters to the original image and the images selected with the application.

Finally, an image modification tool (in this case, GIMP (https://docs.gimp.org/2.10/es/filters.html, accessed on 19 October 2021) was used to generate a set of images with different filters from a single picture. Afterwards, **Aesthetic Selector** just kept those modified images that had improved aesthetic quality. With this, the application can be used as an intelligent selector of filters, able to select those modifications that improve the attractiveness of an image, as we can see in Figure 10.

The application includes one tab completely devoted to interpretability. In this part of the application, one image from the device can be uploaded. Then, the user needs to select one of the two described techniques: smooth grad saliency or GradCAM++. The result of applying that technique will then be shown for the two class labels (“professional shot”/“snapshot”) using the Bernoulli model. This allows visualizations and easy detection of which areas from our input image more strongly influenced the classification of the model.

As a final note, this application is available for Mac OS (>10.13–High Sierra) and Windows 10. As it was developed in Python, it is possible to download the code and run it on any other system with Python 3.6 or a higher version. For more information, visit https://github.com/ferrubio/AestheticSelector, accessed on 19 October 2021.

## 6. Discussion

Regarding the methods proposed in Section 3, the most popular approaches focused on transfer learning were analyzed. In the first experiment, *ConvNet features* were extracted, which were subsequently used as the input in other classification models, converting the activations of the networks into image descriptors. It is true that, at first, some of these descriptors presented a decent behavior comparable to the state-of-the-art techniques. However, due to the large size of some of these descriptors and the limitations of some of the selected classifiers, we saw that the results were surpassed by alternatives based on *fine-tuning*.

In the second experiment, a deep analysis of the most important strategies based on *fine-tuning* was presented. The models implemented in this work, as well as the proposed configurations rivaled the results presented in the original papers. In addition, we reduced the size of some pretrained networks, in this case AlexNet and VGG16, without losing effectiveness in solving the problem of aesthetic quality. We saw how the same results were obtained from networks with a 10% of the size of the original proposal. This reduces the requirements for the evaluation of new cases, allowing such models to be used on devices such as mobile phones or, for example, a Raspberry Pi.

There are several remarkable conclusions we can draw from all of these studies:

- *Fine-tuning* techniques currently presented the best results in the problem of evaluating aesthetic quality in photography;
- The alternatives to the binary classification (NIMA and Bernoulli) presented a promising line of research, since when comparing the results with the rest, it can be seen that, in terms of the accuracy and AUC, the performance was better. In addition, they provided us with predictions about the aesthetics score, so that this information can be used to obtain other metrics;
- With regard to the pretrained ImageNet models used for *fine-tuning*, it was observed that there was hardly any difference between them, so that the networks were already complex enough for a correct interpretation of the images. For this reason, future works should focus on how to represent the output, which is what really produced the variations in the results;
- All the models seen in this work focused on using the votes’ mean to give a quality value and to obtain the results. This is one of the main limitations that we found both in the published works and in this one, since perhaps the average is not the best way to synthesize the voting information.

## 7. Conclusions

The main reason for having carried out such an in-depth study of the methods for solving the aesthetic quality was to obtain a functional application capable of helping both professional and amateur photographers. **Aesthetic Selector** incorporates the models obtained from the experimentation, more concretely our implementation of Bernoulli fine-tuned from Inception. Besides, three possible uses of this tool were already explained and shown in Section 5, which clearly demonstrated its utility. Finally, a first attempt to interpret the decisions of the Bernoulli model with smooth grad saliency and GradCAM++ was shown in Section 4. These tools were added successfully to **Aesthetic Selector**, making it a very useful application even for researchers. We also plan to extend our application with different explainer tools, such as integrated gradients [45] or Shapley additive explanations (SHAP) [46].

It is important to highlight the great effort dedicated to comparing our wok with other works such as NIMA or A-Lamp, obtaining multiple metrics from those models. Above all, the limitation found in the use of the average of the votes as the aesthetics score should also be taken into account. This seems to be one of the key points to be analyzed in future works, where obtaining more informed values from votes will be crucial to improve the results of the models.

As future work, different modifications to the output of neural networks will be carried out in combination with *fine-tuning* processes on ImageNet. A further optimization is also planned by reducing the number of nodes in the last layers of the neural networks. In addition to the memory savings this reduction represents, it allows extracting *ConvNet features* with a smaller dimensionality, but with the same descriptive capacity. This will facilitate exploring the behavior of these image descriptors in other models. Although at first, we did not obtain the expected results, we believe that some probabilistic classifiers can provide good solutions, as these models naturally handle uncertainty and are specially useful in problems where the class is not defined. 

## Figures and Tables

**Table 1:** Summary data of the most important image aesthetic quality datasets.

	No. of Images	Class
PhotoNet	20,278	[1–7]
DPChallenge	16,509	[1–10]
AVA	255,353	[1–10]

**Table 2:** Summary data of the AVA dataset.

	# of Images with the Mean	# of Votes
in the range [1, 2)	5	688,061
in the range [2, 3)	533	1,456,680
in the range [3, 4)	7442	3,566,048
in the range [4, 5)	66,053	8,910,475
in the range [5, 6)	131,166	15,480,896
in the range [6, 7)	46,858	12,204,029
in the range [7, 8)	3249	6,276,270
in the range [8, 9)	47	3,039,389
in the range [9, 10]	0	2,050,983

**Table 3:** Name and number of nodes of the layers from ImageNet-pretrained networks for *ConvNet features* extraction.

Network	Layer (In Keras)	No. of *ConvNet* *Features*
VGG16	fc1	4096
VGG16	fc2	4096
InceptionV3	avg_pool	2048
ResNet50	avg_pool	2048
MobileNet	global_average_pooling2d_1	1024

**Table 4:** Results of three classifiers using *ConvNet features* and general descriptors of the image compared to the state-of-the-art. The best results in each column are bold.

Model (Descriptor)	Accuracy	Balanced Accuracy	AUC
Murray et al. [10]	0.6670	-	-
Kao et al. [33] (AlexNet${}_{fc7}$)	**0.6933**	-	-
Rubio et al. [37] (AlexNet${}_{fc6}$)	0.6350	0.6350	0.6350
Rubio et al. [37] (AlexNet${}_{fc7}$)	0.6450	0.6450	0.6450
Rubio et al. [37] (ResNet-152)	0.6700	**0.6700**	0.6700
Naive Bayes (Color Hist)	0.640	-	0.5300
Naive Bayes (Gray Hist)	0.6450	-	0.5270
Naive Bayes (GIST)	0.5950	-	0.5550
Naive Bayes (Centrist)	0.5500	-	0.5700
Naive Bayes (PHOG)	0.6470	-	0.5600
SVM (Color Hist)	0.5200	-	0.5470
SVM (Gray Hist)	0.4890	-	0.5390
SVM (GIST)	0.5950	-	0.6100
SVM (Centrist)	0.6050	-	0.6050
SVM (PHOG)	0.5750	-	0.5950
Naive Bayes (MobileNet)	0.6467	0.6274	**0.6732**
Naive Bayes (Inception)	0.6221	0.5967	0.6349
Naive Bayes (VGG16${}_{fc1}$)	0.2988	0.5027	0.6285
Naive Bayes (VGG16${}_{fc2}$)	0.3051	0.5063	0.6222
Naive Bayes (ResNet50)	0.3616	0.5138	0.5461
SVM (MobileNet)	0.6576	0.5542	0.5542
SVM (Inception)	0.6392	0.5542	0.5542
SVM (VGG16${}_{fc1}$)	0.6143	0.5209	0.5209
SVM (VGG16${}_{fc2}$)	0.6192	0.5298	0.5298
SVM (ResNet50)	0.6628	0.5113	0.5113
ELM (MobileNet)	0.6881	0.5798	0.6409
ELM (Inception	0.6555	0.5694	0.6205
ELM (VGG16${}_{fc1}$)	0.6645	0.5794	0.6332
ELM (VGG16${}_{fc2}$)	0.6697	0.5642	0.6219
ELM (ResNet50)	0.6802	0.5071	0.5471

**Table 5:** Number of parameters (in millions) of different networks and the memory space required (MB) to store them, depending on the size of the dense layers.

Model	Fully Connected Nodes	Network Params	Model Size
AlexNet	4096	60 M	223 MB
	1000	14 M	54 MB
	500	8.6 M	33 MB
	250	6.1 M	24 MB
VGG16	4096	134 M	513 MB
	1000	41 M	156 MB
	500	28 M	106 MB
	250	21 M	81 M

**Table 6:** Performance of the fine-tuning methods with various architectures in predicting AVA quality compared to the state-of-the-art. Reported accuracy, balance accuracy, and AUC values are based on the classification of photos to a binary class. The mean squared error (MSE), Pearson correlation coefficient (PCC), and Spearman’s rank correlation coefficient (SRCC) are computed between predicted and ground truth mean scores. EMD measures the closeness of the predicted and ground truth rating distributions.

Task	Classification			Score Regression			Distribution Prediction
**Metrics**	**Accuracy**	**Balanced** **Accuracy**	**AUC**	**SRCC**	**PCC**	**MSE**	**EMD**
Murray (AVA) [10]	0.670	-	-	-	-	-	-
Rapid [18]	0.745	-	-	-	-	-	-
DAN1 (AlexNet) [19]	0.713	0.680	-	-	-	-	-
DAN1 (VGG16) [19]	0.741	**0.728**	-	-	-	-	-
DAN2 [19]	0.787	0.695	-	-	-	-	-
NIMA (VGG16) [20]	0.806	-	-	0.592	0.610	-	0.052
NIMA (Inception-v2) [20]	0.815	-	-	0.612	0.636	-	**0.050**
NIMA (ResNet50) [20]	0.793	-	-	0.690	0.694	-	0.067
A-Lamp [42]	0.825	-	-	-	-	-	-
MP-Ada [44]	**0.830**	-	-	-	-	-	-
Bernoulli (VGG16) [43]	0.798	-	-	0.699	0.701	0.289	0.067
Bernoulli (ResNet50) [43]	0.804	-	-	0.714	0.716	0.276	0.066
Bernoulli (ResNet101) [43]	0.808	-	-	**0.719**	**0.720**	**0.275**	0.065
Our DAN1 (AlexNet${}_{4096}$)	0.700	0.690	0.760	-	-	-	-
Our DAN1 (AlexNet${}_{1000}$)	0.700	0.670	0.740	-	-	-	-
Our DAN1 (AlexNet${}_{500}$)	0.690	0.670	0.730	-	-	-	-
Our DAN1 (AlexNet${}_{250}$)	0.700	0.660	0.730	-	-	-	-
Our DAN1 (VGG16${}_{4096}$)	0.700	0.710	0.790	-	-	-	-
Our DAN1 (VGG16${}_{1000}$)	0.720	0.710	0.790	-	-	-	-
Our DAN1 (VGG16${}_{500}$)	0.740	0.710	0.790	-	-	-	-
Our DAN1 (VGG16${}_{250}$)	0.720	0.710	0.790	-	-	-	-
Our NIMA (MobileNet)	0.775	0.670	0.794	0.601	0.595	0.330	0.073
Our NIMA (Inception)	0.795	0.717	0.835	0.668	0.671	0.290	0.067
Our NIMA (ResNet50)	0.777	0.684	0.808	0.611	0.622	0.330	0.071
Our A-Lamp (MobileNet)	0.772	0.647	0.622	0.588	0.596	0.350	-
Our A-Lamp (Inception)	0.783	0.692	0.816	0.622	0.627	0.330	-
Our A-Lamp (ResNet50)	0.781	0.699	0.814	0.619	0.622	0.330	-
Our Bernoulli (MobileNet)	0.774	0.668	0.803	0.597	0.612	0.330	-
Our Bernoulli (Inception)	0.793	0.720	**0.838**	0.664	0.678	0.290	-
Our Bernoulli (ResNet50)	0.776	0.670	0.806	0.602	0.595	0.340	-

**Table 7:** Performance of the Bernoulli and NIMA methods with the Inception architecture fine-tuned with image subsets from AVA. Reported accuracy, balance accuracy, and AUC values are based on the classification of photos to a binary class. MSE and SRCC are computed between predicted and ground truth mean scores.

Task	Classification			Score Regression	
**Metrics**	**Accuracy**	**Balanced Accuracy**	**AUC**	**SRCC**	**MSE**
**Our Bernoulli (Inception)**					
full dataset	0.793	0.720	0.838	0.664	0.290
1/2 dataset	0.787	0.703	0.824	0.639	0.309
1/4 dataset	0.773	0.661	0.804	0.596	0.337
1/8 dataset	0.737	0.573	0.728	0.442	0.490
**Our NIMA (Inception)**					
full dataset	0.795	0.717	0.835	0.668	0.290
1/2 dataset	0.785	0.700	0.823	0.636	0.309
1/4 dataset	0.779	0.686	0.809	0.603	0.333
1/8 dataset	0.737	0.563	0.745	0.480	0.423

## Data Availability

The main dataset presented in this study is available in article Murray, N.; Marchesotti, L.; Perronnin, F. AVA: A large-scale database for aesthetic visual analysis. In Proceedings of the 2012 IEEE Conference on Computer Vision and Pattern Recognition (CVPR), Providence, RI, USA, 16–21 June 2012; IEEE,2012; pp. 2408–2415. Deep learning models developed in this work, with their corresponding weights, are available at https://github.com/ferrubio/AestheticSelector, accessed on 19 October 2021.
